# The Effects of Montelukast on Antioxidant Enzymes and Proinflammatory Cytokines on the Heart, Liver, Lungs, and Kidneys in a Rat Model of Cecal Ligation and Puncture–Induced Sepsis

**DOI:** 10.1100/tsw.2011.122

**Published:** 2011-07-07

**Authors:** Ali Kagan Coskun, Murat Yigiter, Akgun Oral, Fehmi Odabasoglu, Zekai Halici, Oner Mentes, Elif Cadirci, Fadime Atalay, Halis Suleyman

**Affiliations:** ^1^ Department of General Surgery, Gulhane Military Medical Academy, Ankara, Turkey; ^2^ Department of Pediatric Surgery, Ataturk University Faculty of Medicine, Erzurum, Turkey; ^3^ Department of Pediatric Surgery, Gulhane Military Medical Academy, Ankara, Turkey; ^4^ Department of Biochemistry, Ataturk University Faculty of Pharmacy, Erzurum, Turkey; ^5^ Department of Pharmacology, Ataturk University Faculty of Medicine, Erzurum, Turkey; ^6^ Department of Pharmacology, Gulhane Military Medical Academy, Ankara, Turkey; ^7^ Department of Pharmacology, Ataturk University Faculty of Pharmacy, Erzurum, Turkey

**Keywords:** TNF-α, IL-6, polymicrobial sepsis, rat, montelukast, oxidative stress

## Abstract

We investigated the potential protective effects of montelukast (MLK) on cecal ligation and puncture (CLP)–induced tissue injury in vital organs — liver, heart, kidneys, and especially lungs — through inhibition of the proinflammatory cytokine response and the generation of reactive oxygen species (ROS) in rats. The rat groups were (1) a 10-mg/kg MLK-treated CLP group; (2) a 20-mg/kg MLK-treated CLP group; (3) a 20-mg/kg MLK-treated, sham-operated group; (4) a CLP control group; and (5) a sham-operated control group. MLK treatment significantly decreased proinflammatory (tumor necrosis factor-alpha, interleukin-6) cytokine levels following CLP. The lipid peroxide level increased in the lung, heart, liver, and kidney tissues after CLP-induced sepsis, and myeloperoxidase activity increased in the lung, heart, and liver tissues. MLK attenuated this elevation in all tissues except the kidney, dose dependently. The glutathione levels and superoxide dismutase activity were significantly increased in the lung, liver, and kidney tissues after MLK treatment. MLK treatment after CLP also potentially reduced mortality. The lung and kidney tissues were the most protected by MLK under sepsis conditions. We can suggest that MLK reverses the systemic inflammatory reaction to polymicrobial sepsis and thereby reduces multiple organ failure.

## INTRODUCTION

Sepsis is one of the most prevalent diseases and one of the main causes of death among hospitalized patients[1,2]. Sepsis, or septic shock, is a complex pathophysiological procedure characterized by profound hypotension, progressive metabolic acidosis, systemic inflammatory response syndrome (SIRS), tissue damage and multiple organ dysfunction syndrome, acute respiratory distress syndrome (ARDS) and/or acute lung injury (ALI), and even death[3]. Increased vascular permeability of the endothelium occurs in multiple organs during sepsis, leading to plasma extravasations and the subsequent bacterial translocation may play an important role in the development of multiple tissue injury[4]. Although the pathophysiology is not well defined, monocytes orchestrate the innate immune response to bacteria by expressing a variety of inflammatory cytokines, particularly early tumor necrosis factor-α (TNF-α) and interleukin-6 (IL-6)[5,6,7], which lead to SIRS, multiple organ failure, and death. One of the important mechanisms in intra-abdominal sepsis–induced tissue damage is the increase in tissue and plasma oxidative stress due to the generation of free radicals[8]. Oxidants are known to play a major role in inflammation and multiple tissue injury[9]. Under normal physiological conditions, a homeostatic balance exists between the formation of oxygen free radicals and their removal by endogenous scavenging antioxidants[10]. During sepsis, oxygen free radicals are overproduced and the natural scavenging mechanisms are unbalanced. These are the processes that are implicated in microvascular dysfunction and followed by organ dysfunction[11]. It has been reported that there has been a dysfunction of vital tissues, such as the liver[12], heart[13], and kidneys[14], during septic shock as well as ARDS and ALI, which are characterized by the accumulation of a large number of neutrophils in the lungs, related with the increased generation of reactive oxygen and nitrogen species (ROS, RNS) as well as proinflammatory cytokines[15]. Traditional treatment models have not successfully managed ARDS and ALI[16], but protective ventilation strategies have helped to improve mortality rates[3].

Montelukast (MLK) is an anti-inflammatory drug with antioxidant properties[17] that interferes directly with leukotriene reception (leukotriene receptor antagonists)[18]. Leukotrienes, derivatives of arachidonic acid, are important mediators of asthma[19] and have been implicated as mediators of the septic process[20]. Both leukotriene pathway modifiers and LTD4-receptor antagonists have been shown to improve asthma control. Therefore, MLK provides a significant improvement in chronic asthmatic symptoms and maintains a highly safe profile[21]. In addition, studies demonstrating the beneficial usage of leukotriene antagonists in septic conditions have been conducted[20]. Sener et al. also suggested that MLK possesses an anti-inflammatory effect on sepsis-induced hepatic and intestinal damage, and protects against oxidative injury by a neutrophil-dependent mechanism. However, they did not investigate the effect of MLK on vital organs, such as the heart, kidneys, and lungs[22].

Therefore, in this study, we induced sepsis/septic shock with cecal ligation and puncture (CLP, a model of polymicrobial sepsis) in rats and hypothesized that MLK could prevent CLP-induced tissue injury in vital organs — liver, heart, kidneys, and especially the lungs — through the proinflammatory cytokine response and the ROS generation triggered by polymicrobial sepsis.

## MATERIALS AND METHODS

### Animals

In the present study, a total of 100 male Wistar rats, weighing 220–240 g, were used for the experiments. They all were obtained from Ataturk University's Experimental Animal Laboratory of Medicinal and Experimental Application and Research Center (ATADEM). Animal experiments and procedures were performed in accordance with the national guidelines for the use and care of laboratory animals, and were approved by Ataturk University's local animal care committee. The rats were housed in standard plastic cages on sawdust bedding in an air-conditioned room at 22 ± 1°C under controlled lighting (14 h light/10 h dark cycle). Standard rat food and tap water were given *ad libitum*.

## Chemicals

All of the chemicals used in our laboratory experiments were purchased from Sigma Chemical (Germany). Montelukast (MLK) (Singulair® tablet) was obtained from Merck Sharp & Dohme (Istanbul, Turkey).

## Experimental Design

The rats were allocated into five groups, each composed of 20 individual rats: (1) a 10-mg/kg MLK-treated CLP group; (2) a 20-mg/kg MLK-treated CLP group; (3) a 20-mg/kg MLK-treated, sham-operated group; (4) a CLP control group; and (5) a sham-operated control group. The groups were housed separately in different cages.

### *Sepsis Model*

A CLP polymicrobial sepsis model was applied to the rats. Polymicrobial sepsis was induced through cecal ligation and a two-hole puncture. Anesthesia was induced through the intraperitoneal administration of thiopental 25 mg/kg. After the abdomen was shaved, the peritoneum was opened. Once the diaphragm exposed the abdominal organs, the cecum was isolated and ligated with a 3/0 silk ligature just distal to the ileocecal valve. Two punctures were made with a 12-gauge needle through the cecum distal to the point of ligation and the cecum was returned to the peritoneal cavity. The abdominal incision was then closed with a 4/0 sterile, synthetic, absorbable suture. The wound was bathed in 1% lidocaine solution to ensure analgesia. The sham-operated groups received laparotomies and the groups' ceca were manipulated, but not ligated or perforated. All of the animals were given 2 mL/100 g body weight of normal saline subcutaneously at the time of surgery and 6 h after the operations for fluid resuscitation. Immediately after the surgical procedure, the rats in the MLK-treated, sham-operated and the MLK-treated CLP groups received 10- or 20-mg/kg doses of MLK, which were administered with an oral gavage suspended in saline. An equal volume of saline was administered to the sham-operated control group and the CLP control group. The rats were deprived of food postoperatively, but had free access to water for 16 h until they were sacrificed.

All five groups were sacrificed with an overdose of a general anesthetic (thiopental sodium, 50 mg/kg). Cardiac blood samples were collected immediately and transferred to the laboratory in order to facilitate the estimation of the inflammatory cytokines, TNF-α and IL-6, and other biochemical parameter levels in the serum (aspartate aminotransferase [AST], alanine aminotransferase [ALT], blood urea nitrogen [BUN], and creatinine). The lungs, livers, kidneys, and hearts of all rats were rapidly removed and washed in ice-cold saline. The organs were labeled and stored at –80°C until the biochemical analyses were conducted. In this experiment, the number of surviving rats was nine in the CLP control group, 13 in the 10-mg/kg MLK-treated sepsis group, 16 in the 20-mg/kg MLK-treated sepsis group, 20 in the sham-operated control group, and 20 in the MLK-treated, sham-operated group. No samples were taken from dead animals for serum examination and biochemistry.

Nine randomly defined animals were selected from each group (nine rats from the CLP control group, nine rats from the MLK-treated CLP groups, nine rats from the sham-operated group, and nine rats from the MLK-treated, sham-operated group) to use for future serum examination and biochemistry experiments.

## TNF-α and IL-6 Cytokine Serum Measurements

Sera from the five rat groups were separated and stored at −80°C until they were thawed for the assay. IL-6 and TNF-α of one sample were measured with highly sensitive ELISA (enzyme-linked immunosorbent assay) kits (Biosource, USA) specifically designed for rat cytokines, as specified in the manufacturer's instructions. Cytokine assays for each animal and its correlated control were run in the same lot.

## Determination of Serum Enzymes

The separated sera were used to determine ALT and AST activity, as well as BUN and creatinine levels, with an autoanalyzer (Comas C-501).

## Biochemical Investigation of Liver, Lung, Kidney, and Heart Tissues

Activities of superoxide dismutase (SOD) and myeloperoxidase (MPO), and amounts of lipid peroxides (LPO) and glutathione (GSH) enzyme levels in the rat liver, lung, kidney, and heart tissues, were determined by means of macroscopic analyses. To prepare the tissue homogenates, the tissues were ground with liquid nitrogen in a mortar. The ground tissues (0.5 g each) were then treated with 4.5 mL of the appropriate buffer. The mixtures were homogenized on ice using an Ultra-Turrax® Homogenizer for 15 min. The homogenates were filtered and centrifuged using a refrigerated centrifuge at 4°C. These supernatants were then used to determine the enzymatic activity. All assays were performed at room temperature in triplicate.

### *Superoxide Dismutase (SOD) Activity*

Measurements were made according to Sun et al.'s method[23]. SOD estimation was based on the generation of superoxide radicals produced by xanthine and xanthine oxidase, which react with nitro blue tetrazolium (NTB) to form formazan dye. SOD activity was then measured at 560 nm by the degree of inhibition of this reaction and was expressed as millimole per minute per milligram of tissue (mmol $min^{-1}$ mg $tissue^{-1}$).

### *Myeloperoxidase (MPO) Activity*

MPO activity was measured according to Bradley et al.'s modified method[24]. The homogenized samples were frozen and thawed three times, and then centrifuged at 1500 g for 10 min at 4°C. MPO activity was determined by adding 100 µL of the supernatant to 1.9 mL of 10 mmol/L phosphate buffer (pH 6.0) and 1 mL of 1.5 mmol/L o-dianisidine hydrochloride containing 0.0005% (wt/vol) hydrogen peroxide. The changes in each sample's absorbance at 450 nm were recorded on a UV–vis spectrophotometer. MPO activity in all tissues was expressed as micromole per minute per milligram of tissue (µmol $min^{-1}$ mg $tissue^{-1}$).

### *Determination of Lipid Peroxidation (LPO)*

LPO in tissue was determined by estimating the level of malondialdehyde (MDA) using the thiobarbituric acid test[25]. The rats' tissues were promptly excised and rinsed with cold saline. To minimize the possibility of the interference of hemoglobin with the free radicals, any blood adhering to the mucosa was carefully removed. The corpus mucosa was scraped, weighed, and homogenized in 10 mL of 100 g/L KCl. The homogenate (0.5 mL) was added to a solution containing 0.2 mL of 80 g/L sodium laurylsulfate, 1.5 mL of 200 g/L acetic acid, 1.5 mL of 8 g/L 2-thiobarbiturate, and 0.3 mL of distilled water. The mixture was incubated at 98°C for 1 h. After the mixture cooled, 5 mL of n-butanol:pyridine

(15:l) was added. The mixture was centrifuged for 30 min at 4000 rpm. The supernatant was measured at 532 nm and a standard curve was obtained using 1,1,3,3-tetramethoxypropane. The recovery was more than 90%. The results were expressed as nanomole of MDA per gram of tissue (nmol MDA g tissue$^{-1}$).

### *Total Glutathione (GSH) Determination*

The amount of GSH in the tissues was measured according to Sedlak and Lindsay's method[26]. The mucosal surface of the tissue was collected by scraping, and was then weighed and homogenized in 2 mL of 50 mM Tris–HCl buffer containing 20 mM EDTA and 0.2 M sucrose, pH 7.5. The homogenate was immediately precipitated with 0.1 mL of 25% trichloroacetic acid, and the precipitate was removed after centrifugation at 4200 rpm for 40 min at 4°C. The supernatant was used to determine GSH using 5,5'-dithiobis (2-nitrobenzoic acid). Absorbance was measured at 412 nm using a spectrophotometer. The results of the GSH levels in the tissues were expressed as nanomoles per milligram of tissue (nmol mg tissue$^{-1}$).

### Statistical Analysis

Data for the serum cytokine levels measured by ELISA were subjected to one-way analysis of variance (ANOVA) using SPSS 13.0 software. Differences among the groups were obtained using the least significant difference option and it was considered to be significant at $p < 0.05$. A statistical analysis of oxidant and antioxidant enzymes was carried out using one-way ANOVA followed by Duncan's multiple range test (DMRT) using the SPSS software package, version 12.00, and it was considered to be significant at $p < 0.05$. All the results were expressed as mean ± SE for nine rats in each group. Significance between mortality rates was determined with the Chi-square test and Fisher's exact test. Survival curves with COX regression analyses were also computed.

## RESULTS

### Mortality

In the sepsis group, 11 (11/20 – 55%) rats died between 12 and 16 h after CLP-induced sepsis, while in the 10-mg/kg MLK-treated sepsis groups, seven (7/20 – 35%) died between 6 and 16 h, demonstrating that the protective effect of 10 mg/kg MLK was not significant ($p = 0.20$). The 20-mg/kg doses of MLK were more protective than the 10-mg/kg doses, for the number of deaths in the 20-mg/kg MLK-treated sepsis group was four (4/20 – 20%) ($p = 0.02$). No mortality was observed in the sham-operated or the MLK-treated control groups ($p = 0.000$) (Fig. 1). At the end of the experiment, there were nine rats in the CLP control group, 13 rats in the 10-mg/kg MLK-treated sepsis group, 16 rats in the 20-mg/kg MLK-treated sepsis group, 20 rats in the sham-operated control group, and 20 rats in the MLK-treated, sham-operated group. We did not utilize samples from the dead animals for serum examination and biochemistry. Thus, we selected nine randomized animals from each group to use for future serum examination and biochemistry experiments (nine rats from the CLP control group, nine rats from the MLK-treated CLP groups, nine rats from the sham-operated group, and nine rats from the MLK-treated, sham-operated group).

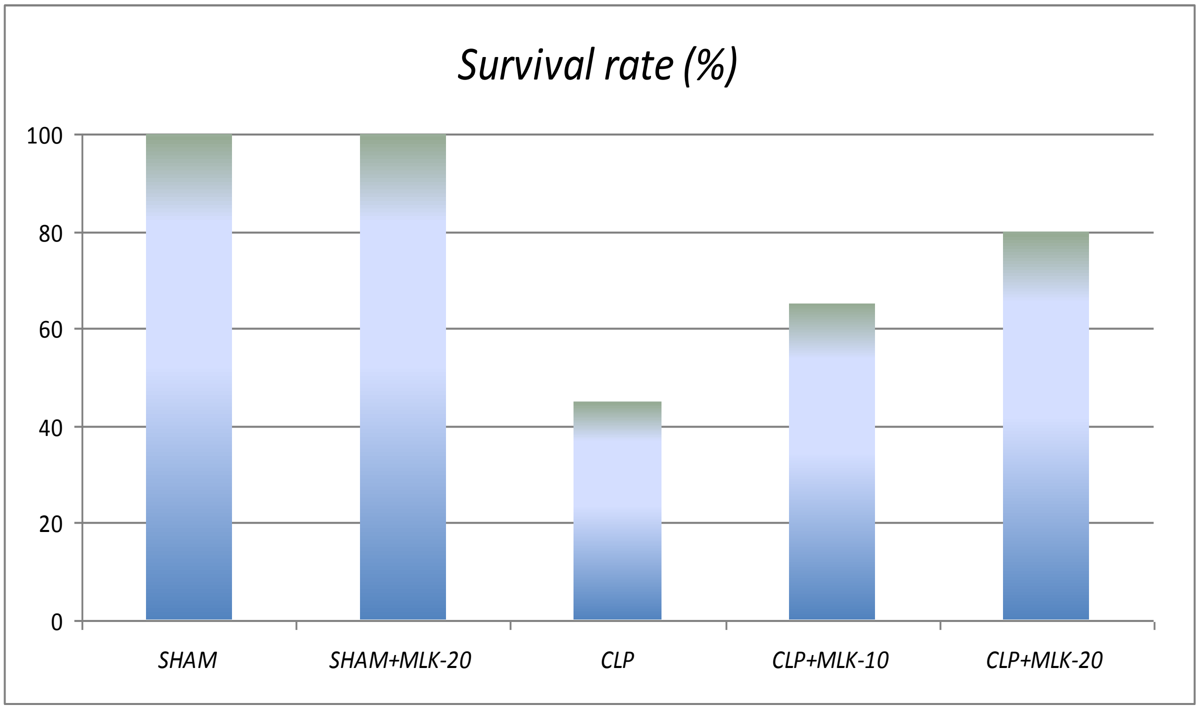

**FIGURE 1.** Survival rates (%) of the experimental groups. CLP: cecal ligation and puncture applied; MLK: montelukast treated; SHAM: sham operated.

## The Effects of MLK on Serum IL-6 and TNF-α Levels in CLP-Induced Septic Rats

In the present study, the serum levels of TNF-α and IL-6, both inflammatory cytokines, were studied in the CLP model based on the sera from the rats (Fig. 2). The levels of both cytokines were found to be increased in the CLP control group (TNF-α: 310.0 ± 20.8 pg/mL; IL-6: 454.6 ± 34.9 pg/mL) when compared with the sham-operated animals (sham group) for which TNF-α was 36.5 ± 7.2 pg/mL and IL-6 was 64.9 ± 13.2 pg/mL, as seen in Fig. 2 ($p < 0.05$). In contrast to the CLP control group, the serum levels of TNF-α and IL-6 were determined to be decreased as a result of MLK administration in the septic rats (MLK-treated CLP groups) ($p < 0.05$). Administration of MLK of 10- and 20-mg/kg doses decreased the serum levels of TNF-α to 198.3 ± 9.3 and 142.5 ± 9.6 pg/mL, respectively ($p < 0.05$). Also, MLK administration of 10- and 20-mg/kg doses decreased the serum levels of IL-6 to 337.3 ± 19.8 and 235.6 ± 21.4 pg/mL, respectively ($p < 0.05$). As can be seen from Fig. 2, the 20-mg/kg dose of MLK was more effective than the 10-mg/kg dose in terms of inflammatory cytokines decrease. Also, the administration of MLK alone in the sham-operated rats did not affect the serum levels of either cytokine (TNF-α: 31.4 ± 7.6 pg/mL; IL-6: 59.0 ± 9.54 pg/mL) when compared to the only sham-operated control group.

## Biochemical Results for Oxidant and Antioxidant Levels of Lung Tissue in Rats

The antioxidant levels (SOD and GSH) were evaluated in all lung tissues. In addition, the levels of oxidant parameters, such as LPO levels and the enzymatic activity of MPO, were also evaluated. The corresponding results are presented in Table 1, showing that SOD activity and GSH levels for the CLP-induced sepsis group were lower, and both MPO and LPO levels were higher than those of the sham-operated rat group ($p < 0.05$). Both doses of MLK had preventive effects on the alterations that occurred in the lung tissues after CLP operation. Both doses of MLK increased SOD activity and GSH levels

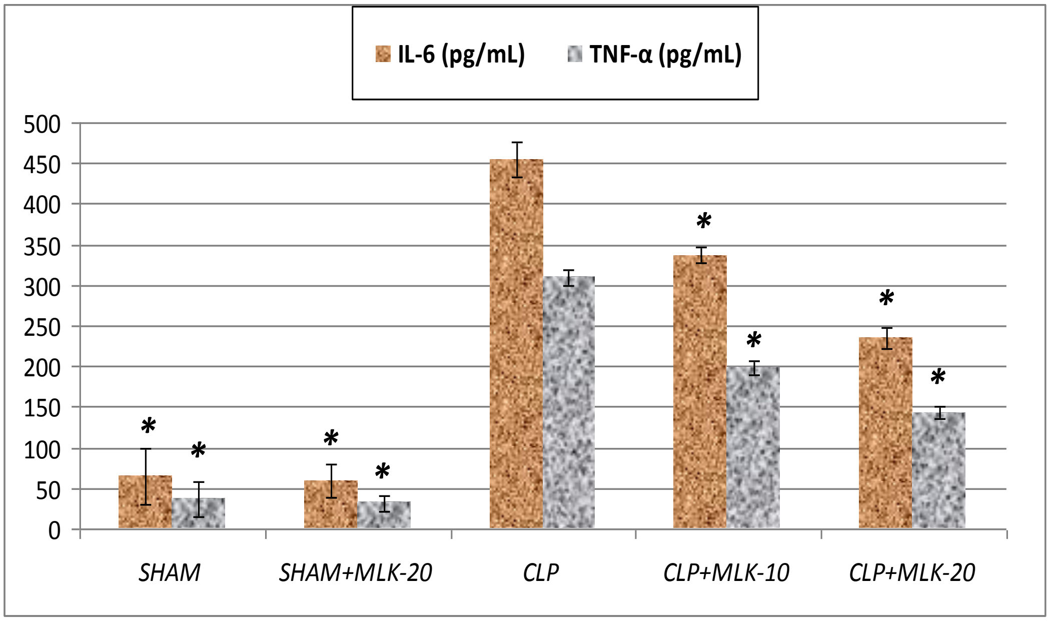

**FIGURE 2.** The effects of MLK on serum IL-6 and TNF-α levels in CLP-induced septic rats. CLP: cecal ligation and puncture applied; MLK: montelukast treated; SHAM: sham operated. * Means significantly different at $p < 0.05$ when compared to CLP control group.

**TABLE 1**
**Effects of MLK Treatments on Changes in Activities of MPO and SOD, and with Levels of LPO and GSH in Lung Tissues of Rats**

| Treatments (Lung Tissue) | N | SOD Activity (mmol/min/mg Tissue) | MPO Activity (µmol/min/mg Tissue) | Amount of LPO (nmol/g Tissue) | Amount of GSH (nmol/mg Tissue) |
|---|---|---|---|---|---|
| SHAM | 9 | 166.1 ± 1.5b | 14.3 ± 0.2c | 14.7 ± 0.4c | 3.5 ± 0.5c |
| SHAM + MLK (20 mg/kg) | 9 | 195.2 ± 1.5d | 9.4 ± 0.2a | 11.3 ± 0.4a | 4.6 ± 0.6d |
| CLP | 9 | 148.8 ± 0.7a | 19.5 ± 0.2e | 18.0 ± 0.1d | 2.8 ± 0.2a |
| CLP + MLK (10 mg/kg) | 9 | 164.7 ± 0.5b | 15.6 ± 0.7d | 14.3 ± 0.2c | 3.3 ± 0.4b |
| CLP + MLK (20 mg/kg) | 9 | 182.8 ± 0.7c | 11.1 ± 0.1b | 13.3 ± 0.1b | 3.6 ± 0.5c |

Means in the same column by the same letter are not significantly different to the Duncan test ($\alpha = 0.05$). Results are means ± SE of measurements. N: the number of rats.

significantly when compared to the CLP control group. The 20-mg/kg dose of MLK also was determined to increase antioxidant levels (SOD and GSH) significantly when compared to the sham-operated group. In addition, both doses of MLK were found to decrease the oxidant parameters significantly when compared to the CLP control group ($p < 0.05$).

## Biochemical Results for Oxidant and Antioxidant Levels of Kidney Tissue in Rats

To explore the effects of antioxidant defenses on the sepsis process, the antioxidant levels (SOD and GSH) were evaluated in all kidney tissues. The levels of oxidant parameters, such as LPO levels and the enzymatic activity of MPO, were also evaluated in all kidney tissues. The corresponding results are presented in Table 2, showing that SOD activity is decreased, while the GSH levels are increased in the CLP-induced sepsis group. Administration of 20 mg/kg MLK to the sham-operated rats increased the levels of both SOD and GSH significantly ($p < 0.05$). Both doses of MLK were determined to have an increasing effect on SOD activity when compared to the CLP control group. Administration of MLK also showed an increased level of GSH significantly when compared to both the sham-operated and the CLP groups ($p < 0.05$). In the kidney tissues of the CLP-induced septic rats, MPO activity decreased significantly compared to the sham group. Administration of MLK to the CLP- and sham-operated rats significantly decreased the MPO activity. The lowest MPO activity was determined in the CLP septic rats treated with 20 mg/kg MLK. Conversely, CLP operation increased the level of LPO in kidney tissue when compared to the sham operation. MLK application was also determined to increase the levels of LPO in kidney tissue ($p < 0.05$).

**TABLE 2**
**Effects of MLK Treatments on Changes in Activities of MPO and SOD, and with Levels of LPO and GSH in Kidney Tissues of Rats**

| Treatments (Kidney Tissue) | N | SOD Activity (mmol/min/mg Tissue) | MPO Activity (μmol/min/mg Tissue) | Amount of LPO (nmol/g Tissue) | Amount of GSH (nmol/mg Tissue) |
|---|---|---|---|---|---|
| SHAM | 9 | 162.8 ± 0.1c | 13.9 ± 0.1e | 14.7 ± 0.2a | 4.0 ± 0.05a |
| SHAM + MLK (20 mg/kg) | 9 | 178.7 ± 0.8d | 11.7 ± 0.2d | 24.0 ± 1.1b | 6.3 ± 0.08c |
| CLP | 9 | 131.9 ± 0.5a | 11.2 ± 0.1c | 37.9 ± 0.3c | 5.0 ± 0.1b |
| CLP + MLK (10 mg/kg) | 9 | 155.2 ± 1.1b | 10.2 ± 0.1b | 40.1 ± 1.2c | 6.1 ± 0.07c |
| CLP + MLK (20 mg/kg) | 9 | 163.3 ± 1.3c | 8.6 ± 0.1a | 47.2 ± 0.7d | 7.0 ± 0.1d |

Means in the same column by the same letter are not significantly different to the Duncan test ($\alpha = 0.05$). Results are means ± SE of measurements. N: the number of rats.

## Biochemical Results for Oxidant and Antioxidant Levels of Liver Tissue in Rats

To explore the effects of antioxidant defenses on the sepsis process, the antioxidant levels (SOD and GSH) were evaluated in all liver tissues. In addition, the levels of oxidant parameters, such as LPO levels and MPO enzymatic activity, were also evaluated in all liver tissues. The results are presented in Table 3, showing that SOD activity increased, but the GSH levels decreased in the CLP-induced sepsis group. Administration of MLK (20 mg/kg) to the sham-operated rats significantly increased the levels of both SOD and GSH compared to the only sham-operated control group. The 10-mg/kg dose of MLK given to septic rats decreased SOD activity significantly compared to the SOD activity of the CLP control group. However, the 20-mg/kg dose of MLK administration increased the SOD activity when compared to the CLP control group. Both doses of MLK increased the GSH levels in septic rats' liver tissue compared to the CLP control group. Both oxidant parameters (MPO and LPO) were found to increase in the livers of the CLP-operated rats when compared to the sham-operated rats. Both doses of MLK significantly decreased the levels of these oxidant parameters in septic rats when compared to the CLP control group. The 20-mg/kg dose of MLK decreased the MPO activity in liver tissue significantly when administered to the sham-operated rats. However, administration of 20 mg/kg MLK did not affect the LPO levels when administered to the sham-operated rats.

**TABLE 3**
**Effects of MLK Treatments on Changes in Activities of MPO and SOD, and with Levels of LPO and GSH in Liver Tissues of Rats**

| Treatments (Liver Tissue) | N | SOD Activity (mmol/min/mg Tissue) | MPO Activity (µmol/min/mg Tissue) | Amount of LPO (nmol/g Tissue) | Amount of GSH (nmol/mg Tissue) |
|---|---|---|---|---|---|
| SHAM | 9 | 116.2 ± 0.2a | 14.4 ± 0.1b | 12.3 ± 0.3a | 3.4 ± 0.1b |
| SHAM + MLK (20 mg/kg) | 9 | 157.7 ± 0.7e | 8.9 ± 0.2a | 11.0 ± 0.4a | 4.3 ± 0.1d |
| CLP | 9 | 137.0 ± 0.8c | 38.2 ± 0.3e | 61.1 ± 0.5d | 3.1 ± 0.1a |
| CLP + MLK (10 mg/kg) | 9 | 133.9 ± 0.5b | 32.9 ± 0.3d | 55.8 ± 0.5c | 3.3 ± 0.1a,b |
| CLP + MLK (20 mg/kg) | 9 | 145.8 ± 0.3d | 27.3 ± 0.6c | 54.0 ± 0.7b | 3.7 ± 0.03c |

Means in the same column by the same letter are not significantly different to the Duncan test ($\alpha$ = 0.05). Results are means ± SE of measurements. N: the number of rats.

## Biochemical Results for Oxidant and Antioxidant Levels of Heart Tissue in Rats

The effects of sepsis and MLK treatment on oxidant and antioxidant parameters were also investigated in the heart tissue of the rats. CLP-induced sepsis significantly increased both the SOD and MPO activity, and the level of LPO when compared to the sham-operated rats. CLP induction had no effect on the GSH level of the heart tissue. Administration of 20 mg/kg MLK to the sham-operated rats significantly decreased SOD, MPO, and LPO when compared to the only sham-operated control group. Administration of the 10-mg/kg dose of MLK to the CLP-operated septic rats significantly decreased SOD and MPO activity, but did not affect the LPO level. However, administration of the 20-mg/kg dose of MLK to septic rats significantly decreased the levels of the three parameters (SOD, MPO, and LPO) when compared to the septic rats on which no MLK medication was applied. Although CLP operation did not affect GSH levels, it is remarkable that the 20-mg/kg dose of MLK decreased the GSH level significantly when administered both to the sham- and CLP-operated rats (Table 4).

**TABLE 4**
**Effects of MLK Treatments on Changes in Activities of MPO and SOD, and with Levels of LPO and GSH in Heart Tissues of Rats**

| Treatments (Heart Tissue) | N | SOD Activity (mmol/min/mg Tissue) | MPO Activity (µmol/min/mg Tissue) | Amount of LPO (nmol/g Tissue) | Amount of GSH (nmol/mg Tissue) |
|---|---|---|---|---|---|
| SHAM | 9 | 142.3 ± 0.4b | 8.4 ± 0.1b | 15.3 ± 0.2b | 3.7 ± 0.1b |
| SHAM + MLK (20 mg/kg) | 9 | 138.1 ± 0.5a | 6.8 ± 0.2a | 13.7 ± 0.1a | 2.4 ± 0.2a |
| CLP | 9 | 157.1 ± 0.2d | 17.1 ± 0.1e | 42.5 ± 0.1d | 3.5 ± 0.1b |
| CLP + MLK (10 mg/kg) | 9 | 147.9 ± 0.7c | 14.5 ± 0.1d | 41.5 ± 0.6d | 3.4 ± 0.2b |
| CLP + MLK (20 mg/kg) | 9 | 138.7 ± 1.0a | 10.8 ± 0.3c | 35.1 ± 0.5c | 2.3 ± 0.1a |

Means in the same column by the same letter are not significantly different to the Duncan test ($\alpha$ = 0.05). Results are means ± SE of measurements. N: the number of rats.

## The Effects of MLK on Serum ALT, AST, BUN, and Creatinine levels in CLP-Induced Septic Rats

As shown in Fig. 3, CLP operation significantly increased the activity of the liver enzymes (AST and ALT) when compared to the sham-operated rats ($p < 0.001$). The AST and ALT levels were determined as 305.1 ± 20.4 and 158.75 ± 14.5 U/L in the CLP control group, while they were 91.5 ± 10.8 and 38.6 ± 8.3 U/L in the sham-operated group. However, both doses of MLK treatment were found to prevent the increase of these enzymes ($p < 0.001$). That is, the serum levels of AST were determined as 194.13 ± 10.3 and 139.5±10.0 U/L, and that of ALT were determined as 93.5 ± 9.6 and 50.1 ± 8.5 U/L for the 10- and 20-mg/kg doses of MLK, respectively. Administration of the 20-mg/kg dose of MLK to the sham-operated rats did not result in a significant difference in the activity of these enzymes (AST: 87.1 ± 15.6 U/L; ALT: 34.9 ± 7.3 U/L) ($p > 0.05$). The effects of CLP on the serum levels of BUN and creatinine are shown in Figs. 4 and 5. In Fig. 4, it can be seen that administration of 20 mg/kg MLK to septic rats produced a significant decrease in BUN levels (29.4 ± 4.8 mg/dL) when compared to the CLP control group, for which it was 60.9 ± 10.2 mg/dL, while the BUN level was significantly increased when compared to the sham group, for which it was 17.5 ± 6.4 mg/dL ($p < 0.001$). The 10-mg/kg dose of MLK also decreased BUN levels significantly ($p < 0.001$) to the level of 39.6 ± 6.5 mg/dL. Similar results were obtained for the creatinine levels, such that the CLP operation increased the serum creatinine level to 1.09 ± 0.12 mg/dL, and both doses of MLK administration reversed the increase in creatinine ($p < 0.001$) to the levels to 0.67 ± 0.5 and 0.46 ± 0.08 mg/dL (Fig. 5). Administration of the 20-mg/kg dose of MLK to the sham-operated rats did not produce significant differences in the serum BUN (19.4 ± 6.6 mg/dL) and creatinine (0.32 ± 0.10 mg/dL) levels when compared to the sham control group, for which the levels of BUN and creatinine were 17.5 ± 6.4 and 0.29 ± 0.11 mg/dL ($p > 0.05$), respectively (Figs. 4 and 5).

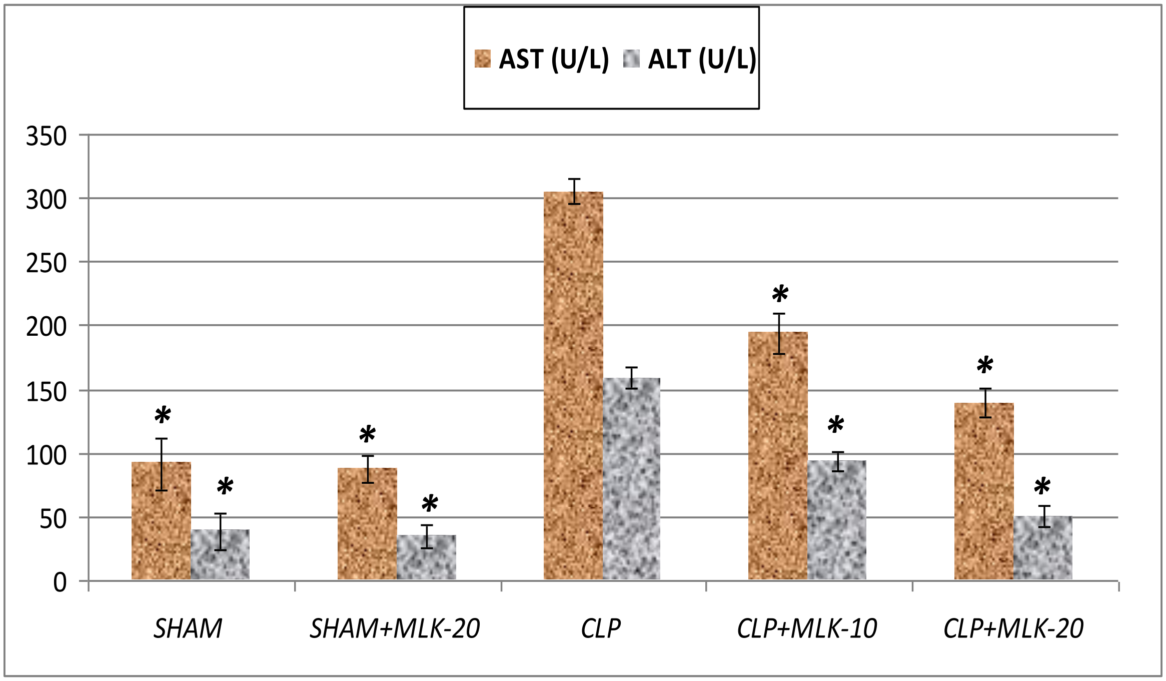

**FIGURE 3.** The effects of MLK on serum AST and ALT activities in CLP-induced septic rats. CLP: cecal ligation and puncture applied; MLK: montelukast treated; SHAM: sham operated. * Means significantly different at $p < 0.05$ when compared to CLP control group.

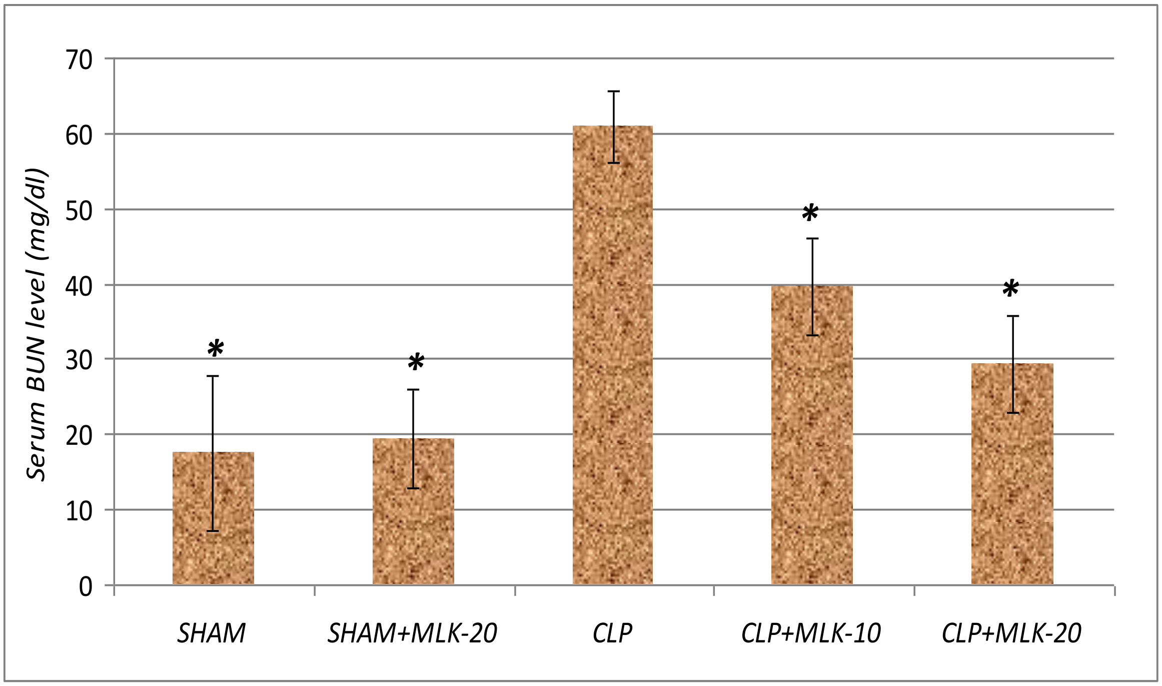

**FIGURE 4.** The effects of MLK on serum BUN levels in CLP-induced septic rats. CLP: cecal ligation and puncture applied; MLK: montelukast treated; SHAM: sham operated. * Means significantly different at $p < 0.05$ when compared to CLP control group.

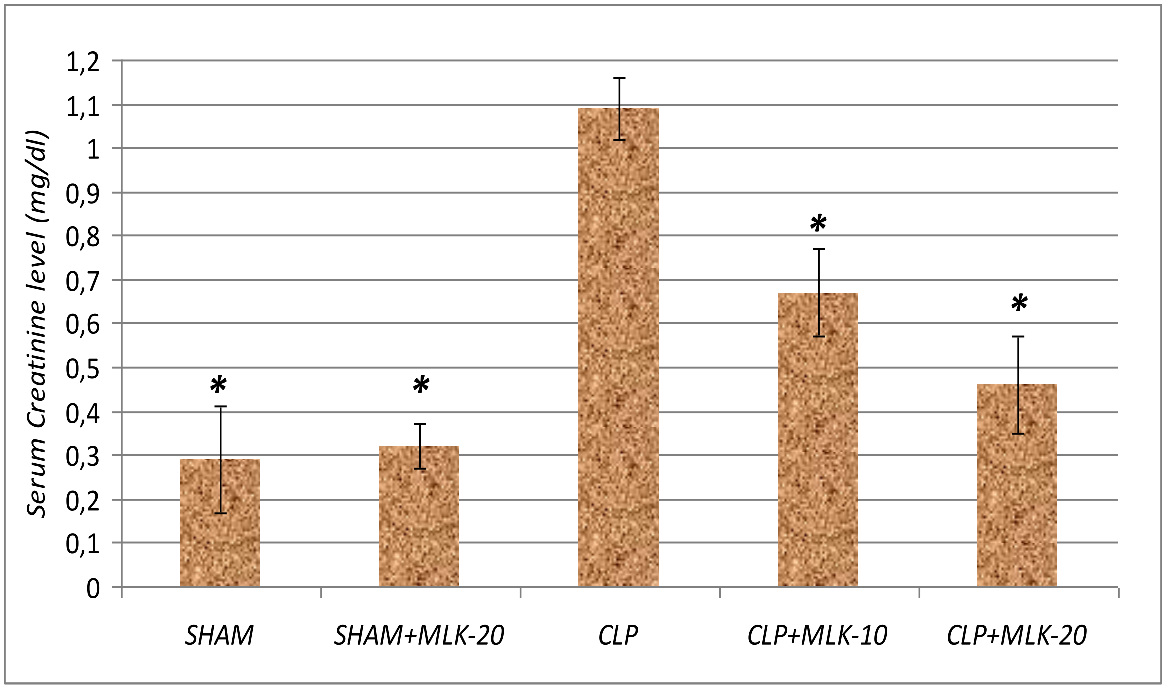

**FIGURE 5.** The effects of MLK on serum creatinine levels in CLP-induced septic rats. CLP: cecal ligation and puncture applied; MLK: montelukast treated; SHAM: sham operated. * Means significantly different at $p < 0.05$ when compared to CLP control group.

## Rat Survival Curve

A survival curve of rats as a function of time is given in Fig. 6. The CLP and 20-mg/kg MLK administered group shows significant results ($p$ = 0.025).

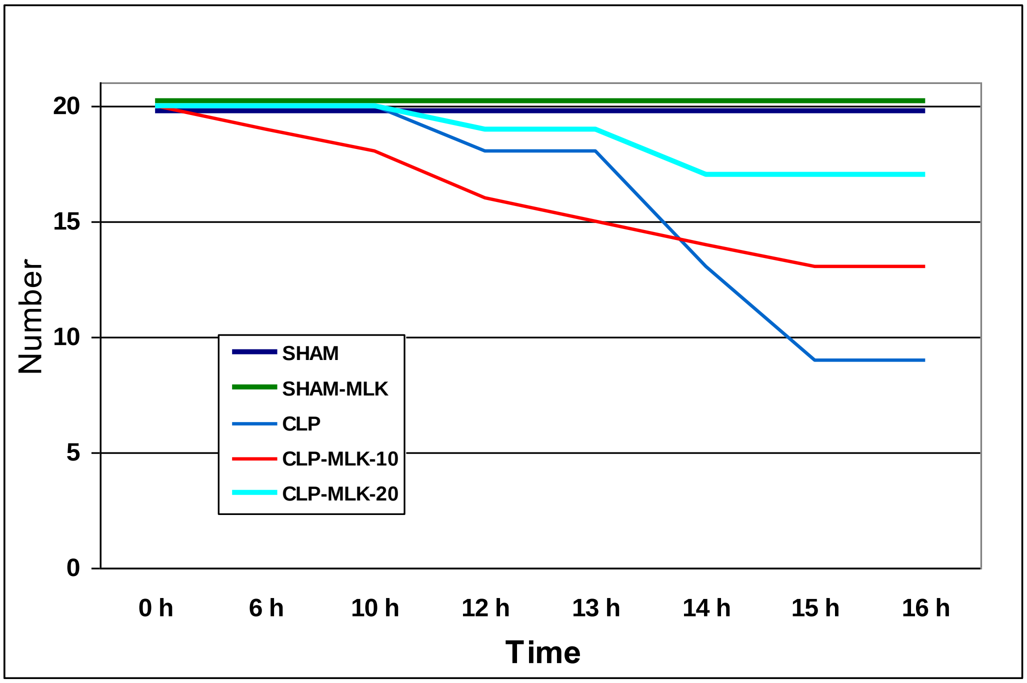

**FIGURE 6.** The survival graphics of the groups.

## DISCUSSION

The effects of MLK on the acute injury of organs such as the kidneys, liver, lungs, and heart induced by polymicrobial sepsis were induced with the previously established CLP model. Fujimura et al.[27] defined a period of over 16 h as the late phase of sepsis. In the current study, we defined the late phase of sepsis at the 16th h. Mortality was high in this sepsis model. In the current study, the mortality rate was 55% at 16 h. However, the rate of mortality was determined to be 20% (4/20) in the 20-mg/kg MLK-treated CLP group. Furthermore, there was no mortality in the sham-operated groups that received MLK or saline.

To the best of our knowledge, we demonstrated the ability of MLK to reduce inflammatory cytokines, such as TNF-α and IL-6, and ameliorate the negative alteration in the tissue levels of SOD, GSH, MPO, and LPO under these conditions. In light of these observations, we can suggest that the therapeutic administration of MLK prevented oxidative stress changes and cytokine changes, and protected vital tissues.

Increased serum early release of proinflammatory cytokines is important in the pathogenesis of septic shock[28], which can be responsible for the ongoing interactions of different cell types, and can aggravate the inflammation and lead to SIRS, multiple organ failure, and death[29]. The cytokines, especially TNF-α, are early regulators of the immune response and can induce the release of secondary cytokines, such as IL-6[30]. TNF-α provokes neutrophil-mediated tissue injury by acting on endothelial cells and neutrophils. A primary role for TNF-α in sepsis is also suggested by several studies conducted on cell lines, animal models, and human beings[15,31,32,33,34,35,36]. TNF-α activates IL-6, which is an important mediator in septic shock and a marker of proinflammatory cytokine activation, and is also defined as an early predictor of organ dysfunction. The measurement of plasma concentration is a valuable predictor for the prognosis of these cases. As such, IL-6 is a useful marker of proinflammatory cytokine activation. Therefore, the TNF-α and IL-6 levels of serum in rats were measured. In this study, the TNF-α and IL-6 levels dramatically increased in the serum of the CLP control group. These results were also supported by data from several other group studies[28,29]. In our study, MLK treatment resulted in significant decreases in proinflammatory (TNF-α, IL-6) cytokine levels following the application of CLP. These data suggest that the ability of MLK given to rats to produce less inflammatory cytokines in response to CLP-induced sepsis may, in part, account for a decrease in cytokine-related organ injury. It seems likely that the anti-inflammatory effect of MLK in CLP-induced sepsis involves the suppression of a variety of proinflammatory mediators produced by the leukocytes and macrophages.

During sepsis, the overproduction of oxygen free radicals and unbalanced natural scavenging mechanisms can cause microvascular dysfunction following organ dysfunction. The accumulation of neutrophils in the lungs, increased generation of ROS/RNS, and increased production of proinflammatory cytokines are main characteristics of organ dysfunction in septic shock which is defined as ARDS and ALI. MLK is an antiasthmatic drug with antioxidant properties[17] that interferes directly with leukotriene production (5-lipoxygenase inhibitors) and/or reception (selective LTD4-receptor antagonists), specifically inhibits the cysteinyl leukotriene (CysLT1) receptor, and reduces airway eosinophilic inflammation[18]. Leukotrienes, derivatives of arachidonic acid, are important mediators of asthma[37] and have been implicated as mediators of the septic process[20]. Sener et al. also demonstrated that MLK ameliorated hepatic and ileal injury in experimental septic conditions[22]. Sepsis and/or septic shock are multisystem diseases with multi–vital organ dysfunction. So it is necessary to investigate all vital organs in order to claim that a treatment protocol is beneficial in septic conditions. The important point about this issue is that some treatment protocols can damage some organs, while protecting the others. Whatever the damage of drugs that has occurred, the main reason for this damage is oxidative stress. The previous study about the effects of MLK on sepsis was focused on only one organ[22], and the evaluation on oxidant and antioxidant parameters was not enough. So we decided to determine effects of MLK in septic conditions by measuring oxidative stress on all vital tissues — the lungs, liver, kidneys, and heart. The overproduction of oxygen free radicals and unbalanced natural scavenging mechanisms can cause microvascular dysfunction following organ dysfunction at sepsis. Increased concentrations of LPO and MPO have been found in rats with sepsis[38], and tissue LPO is a marker of LPO levels that can cause changes in membrane fluidity and permeability, and thus increase the rate of protein degradation, which will eventually lead to cell lysis[39]. In addition, MPO is a marker of neutrophil accumulation[40]. When neutrophils are stimulated, MPO as well as other tissue-damaging substances are responsible for excessive production of ROS such as superoxide ($O^{2-}$)[41]. In our study, LPO activity increased in the lung, heart, liver, and kidney tissues after CLP-induced sepsis, and MPO activity increased in the lung, heart, and liver tissues, but MLK attenuated this elevation in all tissues dose dependently. However, in the kidney tissues, MLK did not decrease the level of increased LPO. Therefore, we thought that MLK may have prevented neutrophil accumulation and LPO in the tissues by decreasing oxidative stress and protecting membrane permeability. Similar results related to the effects of MLK on LPO and MPO have been reported in the literature[22].

In parallel to increases in the MPO and MDA levels, the GSH levels were decreased in the lung, heart, and liver tissues, while increased in the kidney tissues. GSH plays an important role in the maintenance of protein and lipid integrity, and provides major protection in oxidative injury against oxidative damage[42].

Carbonell et al. showed that depletion of liver GSH potentiated the oxidative stress induced by endotoxins in rats, in which plasma LPO levels were raised[43]. In the present study, the levels of GSH were significantly decreased in the lung and liver tissues studied, while increased in the kidney tissue. We suggest that the increase in the GSH levels in the kidney tissue may be a response of the kidney tissue as an augmentation of antioxidant defense mechanisms against increased oxidative stress. Administration of MLK immediately after the CLP procedure increased the lung, liver, and kidney GSH levels. These results suggested that MLK attenuated the oxidative organ injury after CLP-induced sepsis by preventing oxidative stress. Liver, lung, and heart LPO, MPO, and GSH concentrations correlated with plasma proinflammatory cytokine concentrations.

It is very well known that SOD and GSH are real indicators of the antioxidant capacity of the body that are involved in protection against the damage caused by oxidative stress. Ritter et al. showed that plasma SOD levels are markers of early mortality in septic rats[44]. This may explain why the SOD activity decreased in the septic rats in our present study. SOD activity increased in the lung, liver, and kidney tissues due to the administration of MLK immediately after the CLP procedure, but in heart tissues, MLK did not increase the activity of the SOD enzyme.

Our data show that polymicrobial sepsis led to the development of hepatic injury in rats. Serum enzymes, such as ALT and AST, were significantly increased in the sepsis group. However, both doses of MLK treatment were found to prevent the increase of these enzymes. Elevation of AST has been reported to be an index of hepatocellular injury in rats, while ALT elevation is more associated with the necrotic state[45]. Our antioxidant and oxidant parameters in liver tissues of the septic rat group treated with 20 mg/kg MLK supported these findings. Administration of 20 mg/kg MLK to septic rats produced a significant decrease in BUN levels when compared to the CLP control group in which the BUN level was significantly increased when compared to the sham group. Similar results have been obtained for the creatinine levels, such that CLP operation increased the serum creatinine level, and MLK administration turned back the increase in creatinine. The results of this study clearly indicated that administration of MLK to septic rats markedly ameliorated the plasma BUN and creatinine levels.

In conclusion, MLK treatment after CLP potentially reduced mortality in experimental sepsis and may exert its effects through reduction of organs' oxidative stress and the decrease in plasma cytokine levels. MLK had a marked effect on attenuating or decreasing the MPO level of lung, liver, heart, and kidney tissues dose dependently, and decreasing the LPO levels of these tissues except kidney tissue. The lung tissue was most protected by MLK under sepsis conditions. The findings of the current study illustrate that the leukotriene receptor antagonist reverses the systemic inflammatory reaction to polymicrobial sepsis and thereby reduces multiple organ failure.

## CONFLICT OF INTEREST STATEMENT

None of the authors has a commercial interest, financial interest, and/or other relationship with manufacturers of pharmaceuticals, laboratory supplies, and/or medical devices, or with commercial providers of medically related services.
